# Intracellular PD-L1: Functions, Regulation, and Therapeutic Implications

**DOI:** 10.7150/ijbs.133650

**Published:** 2026-07-13

**Authors:** Youliang Zhao, Yaqian Qu, Changfu Hao, Wu Yao

**Affiliations:** 1 Department of Occupational Health and Occupational Disease, College of Public Health, Zhengzhou University, Zhengzhou, Henan, China.; 2 School of medicine, Henan University of Traditional Chinese Medicine, Zhengzhou, Henan, China.

**Keywords:** PD-L1, immune checkpoint, subcellular compartmentalization, posttranslational regulation, cancer immunotherapy

## Abstract

Programmed death-ligand 1 (PD-L1) has long been characterized as a membrane-bound immune checkpoint ligand that suppresses antitumor immunity through engagement with PD-1 on T cells. This canonical understanding has underpinned the development of therapeutic antibodies that have revolutionized cancer treatment. However, PD-1/PD-L1 immunotherapy still has limitations such as poor response rates and post-treatment resistance. Notably, emerging evidence reveals that the roles of PD-L1 extend beyond its membrane-bound form, with substantial pools residing in the cytoplasm, nucleus, organelles, and extracellular vesicles. These intracellular PD-L1 populations perform distinct, often immune-independent functions including transcriptional regulation, mRNA stability control, DNA damage response modulation, and metabolic reprogramming. This review examines the subcellular localization of PD-L1, the mechanisms governing its trafficking and compartmentalization, its compartment-specific biological functions, and the corresponding clinical significance. Understanding the full spectrum of PD-L1 biology is essential for developing more effective immunotherapeutic approaches and promoting individualized treatment strategies.

## 1. Introduction

The discovery that tumors exploit immune checkpoint pathways to evade immune surveillance has transformed cancer treatment. Central to this paradigm is programmed death-ligand 1 (PD-L1), a type I transmembrane glycoprotein of the B7 family that serves as the primary ligand for the inhibitory receptor PD-1 1. When PD-L1 on tumor cells or antigen-presenting cells engages PD-1 on activated T cells, the resulting signaling cascade leads to the suppression of T-cell proliferation, cytokine production, and cytotoxic function. While this mechanism normally maintains immune homeostasis and prevents autoimmunity, cancer cells exploit it to evade immune destruction 2, 3.

The clinical translation of PD-1/PD-L1 blockade represents one of the most significant advances in cancer therapeutics. Multiple monoclonal antibodies targeting this axis (pembrolizumab, nivolumab, atezolizumab, and durvalumab) have received regulatory approval and have demonstrated remarkable efficacy in various cancer types 1, 4. Despite this success, overall response rates rarely exceed 40%, and many patients develop resistance following the initial response 5-7. These limitations highlight fundamental gaps in our understanding of PD-L1 biology that current therapeutic approaches fail to address. Recent discoveries have fundamentally challenged the membrane-centric view of PD-L1. Rather than being confined to the cell surface, PD-L1 has been detected in multiple subcellular compartments, including the endoplasmic reticulum (ER), Golgi apparatus, cytoplasm, nucleus, mitochondria, and extracellular vesicles (EVs), where it performs functions entirely distinct from its canonical immune checkpoint role 8, 9. Intracellular PD-L1 is involved in transcriptional regulation, the stabilization of mRNAs encoding DNA damage response proteins, the modulation of cell cycle progression, and the promotion of metabolic reprogramming 10, 11. These immune-independent activities suggest that targeting membrane-bound PD-L1 alone may be insufficient to fully abrogate PD-L1-mediated tumor progression and therapy resistance.

The recognition that PD-L1 functions as a multifunctional protein across diverse cellular compartments represents a paradigm shift with profound therapeutic implications. This review examines the emerging biology of intracellular PD-L1, encompassing its subcellular distribution, trafficking mechanisms, compartment-specific functions, and regulatory networks that govern its localization. Understanding this complexity is essential for developing next-generation immunotherapeutic approaches and identifying biomarkers that capture the full spectrum of PD-L1 biology.

## 2. Classical Paradigm: Membrane-Bound PD-L1

### 2.1 Structure and Expression of Membrane PD-L1

PD-L1 is a single-pass transmembrane protein comprising a 220-amino acid ectodomain (IgV-like domain spanning residues 19-127 and an IgC-like domain from residues 133-225), a transmembrane domain (residues 239-259), and a 31-amino acid C-terminal cytoplasmic tail (residues 260-290) 4, 8, 12. The protein anchors to the plasma membrane through electrostatic interactions between positively charged residues in the cytoplasmic domain and membrane phospholipids 13. This structural organization positions the IgV-like domain—which mediates PD-1 binding—on the extracellular surface while enabling intracellular signaling through the cytoplasmic tail 14. Under physiological conditions, PD-L1 expression serves critical homeostatic functions across diverse tissues. Its expression has been documented in both hematopoietic cells (T cells, B cells, macrophages, and dendritic cells) 15-18 and nonhematopoietic cells (fibroblasts, endothelial cells, and epithelial cells) 19-21. In malignancy, PD-L1 expression is frequently upregulated on both tumor cells and stromal populations—including tumor-associated macrophages, neutrophils, and cancer-associated fibroblasts—with high expression commonly associated with poor prognosis 22-25.

### 2.2 Canonical PD-1/PD-L1 Signaling

The primary function of membrane-bound PD-L1 involves engaging PD-1 on activated T cells at immune synapses. Upon PD-L1/PD-1 engagement, conformational changes in PD-1 trigger phosphorylation of its immunoreceptor tyrosine-based inhibitory motif (ITIM) and immunoreceptor tyrosine-based switch motif (ITSM) by Src family kinases 26, 27. These phosphorylated motifs recruit the protein tyrosine phosphatases SHP-1 and SHP-2, which dephosphorylate downstream kinases, including ZAP70 and PI3K/AKT 28, 29. The resulting signaling cascade blocks T-cell activation through the PKC, ERK, PLCγ, and RAS pathways, ultimately inhibiting proliferation, reducing cytokine production, and promoting apoptosis 30, 31.

Cancer cells exploit the PD-1/PD-L1 axis as a primary immune evasion mechanism. Constitutive PD-L1 expression provides continuous immune protection, while Interferon gamma (IFN-γ) secreted by tumor-infiltrating lymphocytes paradoxically induces PD-L1 expression on tumor cells—an adaptive resistance mechanism that neutralizes the very immune response toward tumor elimination. This negative feedback loop represents a fundamental mechanism of acquired resistance to immunotherapy 31, 32. In addition, PD-1 expression is transiently upregulated upon T-cell activation; however, its sustained, high-level expression in the context of chronic antigen stimulation is a hallmark of T-cell exhaustion, which is marked by the progressive loss of effector functions 33, 34. In human malignancies, PD-1-positive tumor-infiltrating lymphocytes are enriched for tumor reactivity but exhibit profoundly impaired cytotoxic capacity 35, 36. Therefore, PD-1/PD-L1 signaling is highly anticipated to be targeted for tumor immunotherapy.

### 2.3 Observations Beyond the Membrane

**Figure 1:**
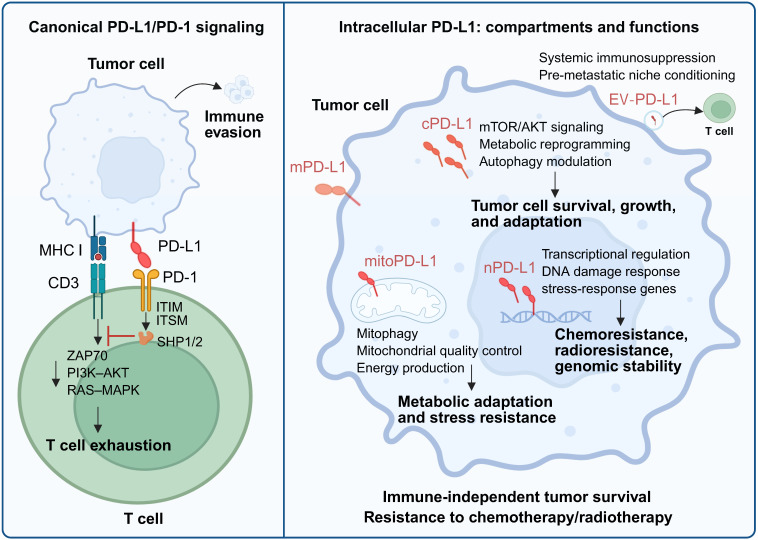
** Canonical PD-L1/PD-1 immune checkpoint activity versus intracellular PD-L1 function.** In the classical view, PD-L1 expressed on tumor cells binds to PD-1 on activated T cells, where it affects immunosuppression signaling. Beyond its role as a surface immune checkpoint, a substantial pool of PD-L1 resides within tumor cells and exerts diverse tumor-regulating effects. Created with BioRender.

Examination of clinical specimens has revealed PD-L1 staining patterns extending beyond the expected membrane localization (Figure 1). Nuclear PD-L1 staining has been reported in cancer tissues, circulating tumor cells, and chemotherapy-treated specimens 37, 38. Similar intracellular localization across multiple cancer types raised questions about whether these patterns represent biologically significant PD-L1 pools or technical artifacts. Moreover, the membrane-centric view of PD-L1 biology cannot fully explain several clinical observations. The poor correlation between single-membrane PD-L1 expression and immunotherapy response suggests that additional factors influence therapeutic outcomes 39. Furthermore, tumor-intrinsic functions of PD-L1—including the promotion of proliferation, drug resistance, and metabolic reprogramming—have been demonstrated in immunodeficient models lacking T cells, indicating PD-1-independent activities 40, 41. These findings suggest that PD-L1 has important functions in intracellular compartments that current antibody-based therapies cannot address. The dynamic distribution of PD-L1 across multiple compartments with distinct functions at each location necessitates a more comprehensive approach. This complexity may explain why existing antibodies that primarily target membrane-bound PD-L1 have limited efficacy in many patients, suggesting that therapeutic strategies must account for noncanonical pathways and their context-dependent effects. The following sections discuss the emerging evidence for the functions of intracellular PD-L1 and its implications for cancer therapy.

## 3. Subcellular Localization of PD-L1

**Figure 2:**
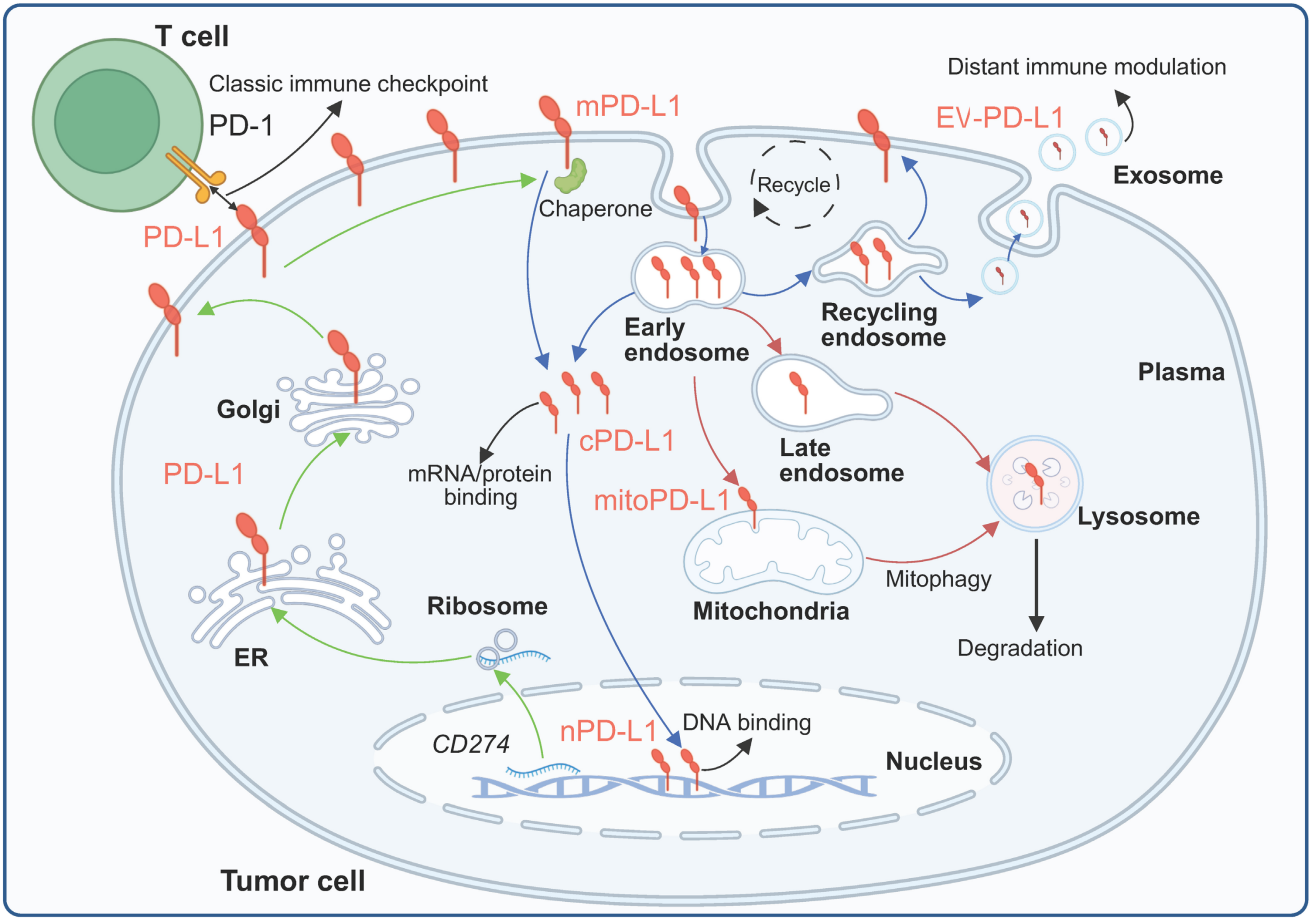
** Subcellular localization and trafficking of PD-L1.** Membrane-localized PD-L1 can enter cells and be distributed to different compartments or further released extracellularly under the synergistic regulation of complex mechanisms. Created with BioRender.

Observations of intracellular PD-L1 were initially met with skepticism, with some investigators arguing that these patterns represented technical artifacts 42. However, studies employing multiple independent fractionation methods with extensive purity validation using different compartment markers (GAPDH, calpain-2, EGFR, Na-K-ATPase, lamin A/C, histone H3, β-actin, and β-tubulin) have demonstrated consistent PD-L1 expression across cytoplasmic, membrane, nuclear soluble, nuclear chromatin-bound, and cytoskeletal fractions 8, 9, 43. This review focuses mainly on PD-L1 in the nucleus, cytoplasm, organelles and EVs (Figure 2).

### 3.1 Cytoplasmic PD-L1

Cytoplasmic PD-L1 expression has been confirmed in both tumor cells and nontumor cells. The cytoplasmic pool represents a dynamic reservoir that can be rapidly mobilized to the cell surface or directed toward degradation pathways depending on cellular signals 8, 44. Cytoplasmic PD-L1 is associated with multiple organelles through biosynthetic and endocytic pathways. APEX2 proximity mapping studies have demonstrated that PD-L1 colocalizes with transferrin receptor and Rab11, which are markers of the recycling endosome compartment 45. Perinuclear staining patterns suggest the presence of this protein in the endosomal recycling compartment, which is consistent with constitutive trafficking through the secretory and endocytic systems 46. PD-L1 is distributed across the endosomal system, with localization depending on the balance between recycling and degradation signals. CKLF-like MARVEL transmembrane domain-containing protein 6 (CMTM6), a critical regulator of PD-L1 stability, is associated with PD-L1 at both the plasma membrane and recycling endosomes, protecting it from lysosomal degradation 47.

### 3.2 Nuclear PD-L1

Nuclear PD-L1 expression has been documented across multiple cancer types. In V-RAF murine sarcoma viral oncogene homolog B (BRAF)-mutated colorectal cancer (CRC), nuclear PD-L1 staining was detected in 72.7% of samples, whereas minimal nuclear staining was detected in BRAF wild-type tumors 38. The expression of nuclear PD-L1 in cell-surface vimentin-positive circulating tumor cells was significantly associated with short-term survival in patients with colorectal and prostate cancer, establishing prognostic significance 48. Although PD-L1 lacks a canonical nuclear localization signal, nuclear import occurs through the classical importin pathway involving KPNA2 and KPNB1 49. In non-small-cell lung cancer (NSCLC), KPNB1 directly binds PD-L1 and promotes its nuclear transport 50. The requirement for the importin machinery suggests that PD-L1 may contain cryptic NLS motifs or require adaptor proteins for nuclear entry. Furthermore, the nuclear translocation of PD-L1 is regulated by a complex molecular network, and nuclear PD-L1 has diverse functions, as detailed below.

### 3.3 Organellar PD-L1

#### 3.3.1 Endoplasmic Reticulum

The ER is a critical checkpoint for PD-L1 quality control, where proper glycosylation is required for forward trafficking 51. Adenosine 5′-monophosphate-activated protein kinase (AMPK)-mediated phosphorylation at S195 induces abnormal glycosylation, causing ER retention and subsequent ER-associated degradation (ERAD) 52. ER chaperones, including the Sigma1 receptor and FKBP51s facilitate PD-L1 folding and glycosylation; inhibition of these chaperones induces PD-L1 degradation through ER stress and autophagy pathways 53.

#### 3.3.2 Golgi Apparatus

The Golgi is the site of complex glycan maturation of PD-L1, with N-acetyllactosaminide beta-1,3-N-acetylglucosaminyltransferase 3 (B3GNT3) mediating poly-N-acetyllactosamine addition, which is essential for PD-1 binding capacity 54. Vacuolar protein sorting-associated protein 18 homolog (VPS18) and VPS11 regulate PD-L1 trafficking through the trans-Golgi network, promoting glycosylation and protein stability 43. Disruption of Golgi processing leads to the accumulation of abnormally glycosylated PD-L1 targeted for degradation.

#### 3.3.3 Mitochondria

Recent evidence has indicated that mitochondrial PD-L1 localization is functional. An ATAD3A-PINK1-mitophagy axis regulates the subcellular distribution of PD-L1, with PINK1 recruiting PD-L1 to mitochondria for mitophagy-mediated degradation. ATAD3A suppresses this process, and its upregulation by paclitaxel disrupts PD-L1 proteostasis, causing excessive cell surface accumulation and immunosuppression 55.

### 3.4 Extracellular vesicle PD-L1

Tumor cells package PD-L1 into EVs through endosomal sorting complexes required for transport (ESCRT)-dependent processes. The ESCRT-0 component HRS is associated with ERK, and ERK-mediated HRS phosphorylation increases EV-PD-L1 levels. ALIX binds the ESCRT-III complex and promotes PD-L1 packaging into EVs in a calcium-dependent manner 56. EV-PD-L1 maintains the same protein structure as its membrane-bound form and retains its immunosuppressive function by binding to PD-1 on effector T cells. Notably, compared with membrane PD-L1, EV-PD-L1 is more stable because of its association with MHC-I molecules and can mediate systemic rather than local immunosuppression 57. Although EVs are not essentially intracellular components once released, we include EV-associated PD-L1 because its biogenesis, cargo sorting, and secretion are entirely dependent on intracellular trafficking and endosomal sorting pathways, and it is in dynamic equilibrium with intracellular PD-L1 pools.

In conclusion, PD-L1 is synthesized in the ER, where it undergoes glycosylation and is then further processed in the Golgi before delivery to the plasma membrane. Membrane PD-L1 is dynamically internalized into the endosomal system, from which it can either be recycled back to the plasma membrane by recycling endosomes, be trafficked to late endosomes/lysosomes for degradation, or be secreted extracellularly within EVs. A substantial amount of the intracellular pool of PD-L1 also localizes to the mitochondria or is translocated to the nucleus, where it performs specific functions.

## 4. Regulatory Mechanisms of Intracellular PD-L1

### 4.1 Transcriptional and Posttranscriptional Regulation

#### 4.1.1 Transcription Factors and Epigenetic Regulators

The transcriptional regulation of PD-L1 involves complex interactions between multiple transcription factors and pathways. The JAK/STAT/IRF1 axis represents the most prominent inducible regulatory pathway of PD-L1. IFN-γ activates JAK/STAT signaling, with signal transducer and activator of transcription 1 (STAT1) upregulating the expression of interferon-responsive factors (IRFs), particularly IRF-1, which bind to IRE1/2 in the CD274 promoter 3, 58. STAT3 also acts directly on the PD-L1 promoter, with some cancers harboring functional STAT3 mutations that induce PD-L1 expression in the absence of cytokines 59. Nuclear factor-κB (NF-κB) translocates to the nucleus upon cytokine stimulation and binds to target sites at the PD-L1 promoter in a pathway activated by tumor necrosis factor-α (TNF-α) and oncogenic mutations 60. Hypoxia induces PD-L1 expression through HIF-1α and HIF-2α, which physically interact with hypoxia response elements in the PD-L1 promoter 61. Oncogenic transcription factors, including MYC, directly bind the PD-L1 promoter, driving immune evasion and contributing to cisplatin resistance 62. Epigenetic mechanisms significantly influence CD274 transcription. DNA methyltransferase inhibitors increase PD-L1 levels by increasing the expression of hypermethylated endogenous retroviruses that activate innate immune responses 63. Histone modifications also regulate expression: MLL1 protein binds to the CD274 promoter to catalyze H3K4 tri-methylation, increasing PD-L1 expression, whereas EZH2-mediated H3K27me3 downregulates expression 64, 65. HDAC inhibitors increase PD-L1 expression by maintaining histone acetylation, and hyperactivated JNK/c-Jun signaling suppresses HDAC3 expression, increasing histone H3 acetylation at the CD274 promoter 66. Overall, PD-L1 expression is extensively regulated at both the transcriptional and epigenetic levels.

#### 4.1.2 miRNA Regulation

PD-L1 mRNA contains an exceptionally long 3′-UTR compared with its relatively short coding sequence, enabling sophisticated posttranscriptional regulation. Multiple miRNAs directly target this region: miR-513 was the first identified miRNA that directly inhibits PD-L1 translation, with its expression inhibited by IFN-γ 67. Other miRNAs, including miR-34a, miR-152, miR-576, miR-15a, miR-197, miR-16, miR-200, miR-20, miR-21, and miR-130b, that directly or indirectly regulate PD-L1 expression have been discussed in other reviews 68.

#### 4.1.3 mRNA Stability

Structural variations in the CD274 3′-UTR significantly impact mRNA stability. The 3′-UTR of PD-L1 contains at least four AU-rich elements (AREs) with a core ATTTA motif; tristetraprolin (TTP) recognizes these AREs and negatively regulates PD-L1 mRNA, whereas activated RAS signaling inhibits TTP via MK2-mediated phosphorylation, rescuing PD-L1 mRNA from degradation 58. In addition, PD-L1 protein expression is induced by the recruitment of polysomes to PD-L1 mRNA in response to PI3K/Akt/mTOR/S6K1 pathway activation, demonstrating translational control beyond transcriptional and mRNA stability mechanisms 69.

### 4.2 Posttranslational Modifications

#### 4.2.1 Glycosylation

N-linked glycosylation profoundly influences PD-L1 stability, trafficking, and function. PD-L1 is heavily glycosylated at four asparagine residues (N35, N192, N200, and N219), with the majority existing as glycosylated forms ranging from 45-55 kDa (compared to 33 kDa nonglycosylated) 70, 71. Glycosylation extends the half-life of PD-L1 by protecting against glycogen synthase kinase-3 beta (GSK3β)-mediated proteasome degradation. The glycosylation process begins in the ER, where a preassembled oligosaccharide is transferred by oligosaccharyltransferase, followed by trimming to generate high-mannose glycans in the ER and complex-type glycans in the Golgi 70. IL-6-activated JAK1 phosphorylates PD-L1 at Y112, recruiting STT3A to catalyze glycosylation 72. B3GNT3 mediates the poly-N-acetyllactosamine addition required for PD-L1/PD-1 binding 73. The chaperone Sigma1 facilitates PD-L1 folding in the ER, which is a prerequisite for glycosylation 53. Conversely, deglycosylation triggers nuclear translocation. Ionizing radiation induces global deglycosylation of PD-L1. Importantly, the specific deglycosylation at residue N219 mediated by NGLY1 was shown to be both necessary and sufficient for its subsequent nuclear translocation 74. Therefore, glycosylation is a key switch for PD-L1 stability and localization, and its dynamic regulation is closely related to the tumor immunotherapy response.

#### 4.2.2 Phosphorylation

Multiple phosphorylation events regulate PD-L1 stability, trafficking, and localization through distinct mechanisms. GSK3β phosphorylates nonglycosylated PD-L1 at T180 and S184, creating a phosphodegron that recruits the E3 ligase β-TrCP via its DSG motif at S176, catalyzing K48 ubiquitination and proteasomal degradation. Glycosylation at N192, N200, and N219 creates spatial hindrance that prevents interaction between GSK3β and PD-L1 71. AMPK-mediated phosphorylation at S195 induces abnormal glycosylation, preventing PD-L1 transport from the ER to the Golgi and triggering ERAD. Mass spectrometry revealed that S195-phosphorylated PD-L1 contains only mannose-rich glycan structures characteristic of ERAD substrates 52. AMPK phosphorylation at S283 in glucose-deprived cells disrupts the PD-L1-CMTM4 interaction, triggering degradation 75. Additional phosphorylation sites include S279/S283 (GSK3α-mediated, promoting ARIH1-mediated ubiquitination when EGFR is inhibited) and T285/T290 (CK2-mediated, disrupting SPOP binding and protecting PD-L1 from CUL3-mediated degradation) 76, 77. NEK2 phosphorylates T194 and T210, promoting glycosylation and stability 78. In summary, the phosphorylation regulation of PD-L1 is intricately regulated at multiple sites and with multiple kinases, suggesting that the selective intervention of phosphorylation events in different tumor states may reverse immune escape.

#### 4.2.3 Ubiquitination

Ubiquitination serves as a major regulatory mechanism controlling PD-L1 abundance and fate. Multiple E3 ubiquitin ligases target PD-L1: For example, β-TrCP mediates K48-linked polyubiquitinnation of phosphorylated nonglycosylated PD-L1 for proteasomal degradation 71. The SPOP-CUL3 pathway recognizes PD-L1 via its C-terminal tail; CDK4 phosphorylates SPOP, disrupting its interaction with CDH1 and preventing APC/Cdh1-mediated SPOP degradation, thereby promoting PD-L1 ubiquitination 77. SPOP loss-of-function mutations increase PD-L1 protein stability, resulting in tumor immunosuppression. ARIH1 mediates K48-linked ubiquitination at S279/S283 when the activity of EGFR is inhibited through GSK3α-mediated phosphorylation 76. STUB1 destabilizes the PD-L1 protein by inducing ubiquitination and lysosomal degradation 79. HRD1 mediates the degradation of abnormally glycosylated PD-L1 in the ER through the ERAD pathway 80. By counteracting ubiquitination, several deubiquitinases stabilize PD-L1. COP9 signalosome complex subunit 5 (CSN5), induced by TNF-α through NF-κB activation, removes ubiquitin chains to prevent degradation 81. USP19 and OTUB1 interact with the PD-L1 cytoplasmic domain to remove ubiquitin modifications 82, 83. CMTM6 and CMTM4 protect PD-L1 from STUB1-mediated polyubiquitination, prolonging its half-life at the plasma membrane and recycling endosomes 47. It is evident that the regulation of PD-L1 ubiquitination is precisely balanced by a variety of E3 ligases and deubiquitination enzymes. This dynamic network provides an interventionable node for tumor immunotherapy.

#### 4.2.4 Acetylation, Methylation, and Other Modifications

Acetylation critically regulates PD-L1 nuclear translocation. p300 acetyltransferase acetylates PD-L1 at K263 in the cytoplasmic domain, while histone deacetylase 2 (HDAC2) mediates deacetylation. Acetylation blocks huntingtin-interacting protein 1-related protein (HIP1R) binding and suppresses nuclear translocation, maintaining PD-L1 at the membrane; deacetylation allows the interaction of HIP1R and triggers its nuclear import. HBXIP facilitates p300-mediated acetylation at K270, stabilizing PD-L1 in breast cancer cells 10, 84. Lactylation is a recently discovered modification: p300 catalyzes lactylation at K189, with HDAC2 serving as the delactylase. K189 delactylation enhances vimentin binding and promotes nuclear import, while lactylation results in the retention of PD-L1 in the cytoplasm. Low K189 lactylation is correlated with high histological grade and poor survival in liver cancer patients 85. Palmitoylation blocks monoubiquitination and inhibits PD-L1 degradation. Palmitoylation at C272, mediated by ZDHHC3 and ZDHHC9, stabilizes PD-L1 by preventing ESCRT-dependent internalization to multivesicular bodies and lysosomes 86 (Table 1). In summary, various protein modification mechanisms that have not yet been fully explored can regulate the intracellular localization and homeostasis of PD-L1, and abnormal modifications are closely related to tumor progression, making them worthy of future research attention.

### 4.3 Trafficking and Subcellular Redistribution

#### 4.3.1 Determinants of ER-to-membrane Progression

Successful ER-to-membrane trafficking requires proper glycosylation and quality control. FKBP51s facilitates PD-L1 folding as a prerequisite for glycosylation; inhibition results in PD-L1 degradation through ER stress and autophagy 87. Sigma1 interacts with both glycosylated and nonglycosylated PD-L1, with primary associations occurring with early glycosylated forms in the ER and early Golgi compartments 53. VPS18 and VPS11 regulate PD-L1 trafficking through the trans-Golgi network, promoting glycosylation and protein stability 43. JAK1-mediated Y112 phosphorylation leads to the recruitment of STT3A to catalyze glycosylation, establishing a link between inflammatory signaling and ER processing 88. These findings suggest that targeting and blocking the maturation of the PD-L1 protein may reverse immunosuppression in its early stages and potentially prevent compensatory resynthesis caused by membrane protein degradation.

#### 4.3.2 Mechanisms of Intracellular Retention

Multiple mechanisms can retain PD-L1 intracellularly rather than allowing surface expression. AMPK-induced S195 phosphorylation triggers abnormal glycosylation, preventing ER-to-Golgi transport and resulting in ERAD 52. Aberrant glycan synthesis leads to HRD1-mediated ERAD-dependent degradation 80. The balance between recycling and degradation pathways also determines intracellular accumulation, with O-GlcNAcylation of the sorting machinery shifting this balance toward recycling and away from degradation 89. Therefore, the dynamic balance of intracellular PD-L1 retention, degradation, and recycling provide a window for precise tumor intervention.

#### 4.3.3 Endocytosis, Recycling, and Degradation Pathways

PD-L1 undergoes constitutive clathrin-dependent endocytosis with a recycling time of approximately 15 minutes, maintaining approximately 70% at the cell surface and 30% in intracellular compartments at equilibrium 10, 90. CMTM6 binds plasma membrane PD-L1 and recycling endosomes, inhibiting endocytosed PD-L1 degradation and enhancing protein stability 47. TRAPPC4 promotes PD-L1 recycling away from the lysosomal pathway. HIP1R directs PD-L1 fate through multiple mechanisms: It contains a lysosomal sorting signal that targets PD-L1 to Rab7 late endosomes and LAMP1 lysosomes for degradation 84. PKCα/GSK3β/MITF pathway activation promotes lysosome biogenesis, leading to autophagic degradation of PD-L1 91. Conversely, C272 palmitoylation prevents lysosomal degradation by blocking ESCRT-dependent internalization 86. Therefore, targeting molecules that mediate PD-L1 recirculation or induce autophagy can directionally clear PD-L1 from tumor cells.

#### 4.3.4 Nuclear Import/Export Machinery

The nuclear translocation of PD-L1 occurs through a multistep process that requires specific protein interactions and posttranslational modifications (PTMs). STT3-dependent N-glycosylation stabilizes the PD-L1 protein and promotes its transport from the ER to the cell membrane, providing a basic substrate for nuclear translocation 72. Following HDAC2-mediated K263 deacetylation, PD-L1 interacts with HIP1R, triggering clathrin-dependent endocytosis 84. Vimentin, an intermediate filament protein, facilitates cytoskeletal trafficking, with the highest PD-L1/vimentin interaction occurring during the G1-to-S transition and interaction decreasing in the G2/M phase as PD-L1 reaches its nuclear destination 48. Here, glycosylation can regulate the membrane retention and endocytosis efficiency of PD-L1, indirectly regulating the modification of PD-L1 by acetylation enzymes. In addition, the acetylation state further determines the efficiency of PD-L1 binding to vimentin and transporting it to the nucleus via the cytoskeleton, ultimately leading to nuclear translocation of PD-L1 through the importin-mediated nuclear import process. Under hypoxic conditions, phosphorylated STAT3 physically interacts with PD-L1 and facilitates nuclear translocation 92. K189 delactylation increases vimentin binding, promoting nuclear import through the same pathway 85. Nuclear PD-L1 undergoes reversible acetylation; acetylation by p300 enables nuclear export via exportin, creating a dynamic shuttle between nuclear and membrane compartments 10. The transport of nuclear PD-L1 is regulated by multiple mechanisms, and its intervention value deserves attention.

#### 4.3.5 Molecular Chaperones and Escort Proteins

In addition to acting as an ER-resident chaperone, CMTM6 serves as a critical escort protein accompanying PD-L1 during trafficking. CMTM6 directly interacts with both glycosylated and deglycosylated PD-L1, and ionizing radiation promotes PD-L1/CMTM6 interactions that occur from the cytoplasm to the nucleus. Silencing CMTM6 blocks IR-induced nuclear PD-L1 translocation, establishing that CMTM6 is essential for stress-induced redistribution 44. Under hypoxia, HIF1α upregulates NMT1, which catalyzes the N-myristoylation of CHP1. Myristoylated CHP1 exhibits enhanced binding affinity to PD-L1, promoting membrane localization 93. This mechanism reveals that intracellular PD-L1 is a dynamic reservoir that can be mobilized to the surface through adaptor protein modifications.

### 4.4 Stimulus-Induced Relocalization

Hypoxia profoundly influences PD-L1 localization through multiple mechanisms. Hypoxia-induced phosphorylated STAT3 physically interacts with PD-L1 and facilitates nuclear translocation 61. In breast cancer under hypoxic conditions, nuclear PD-L1 enhances GSDMC gene transcription, with GSDMC cleavage by caspase-8 generating a pyroptosis-inducing N-terminal domain 92. Hypoxia-induced perturbations in acetyl-CoA synthase impact PD-L1 acetylation, suggesting that the intricate interplay between hypoxia and acetylation serves as a potential initiating factor for nuclear PD-L1 transport 94. IFN-γ is the most potent inducer of PD-L1 expression and promotes time-dependent nuclear PD-L1 accumulation. Nuclear PD-L1 is then recruited to the promoters of genes, including IL-8, Bcl3, and STAT1, creating a positive feedback loop in which induced nuclear PD-L1 further enhances the transcription of inflammatory genes 95. TNF-α increases CD274 mRNA expression by activating NF-κB, which also induces CSN5 expression to inhibit PD-L1 ubiquitination and degradation 96. Metabolic stress signals directly regulate intracellular PD-L1. Energy deprivation activates AMPK, which phosphorylates PD-L1 at S195 and S283. S195 phosphorylation induces abnormal glycosylation and ERAD, whereas S283 phosphorylation disrupts CMTM4-mediated protection, leading to PD-L1 degradation 52, 75. DNA damage is a potent inducer of PD-L1 relocalization. Ionizing radiation induces PD-L1 deglycosylation and triggers its nuclear translocation 74. Nuclear PD-L1 then interacts with Ku80 to enhance nonhomologous end joining (NHEJ) mediated DNA repair. Doxorubicin redistributes PD-L1 and increases nuclear PD-L1 expression through PI3K/Akt signaling 97.

**Figure 3:**
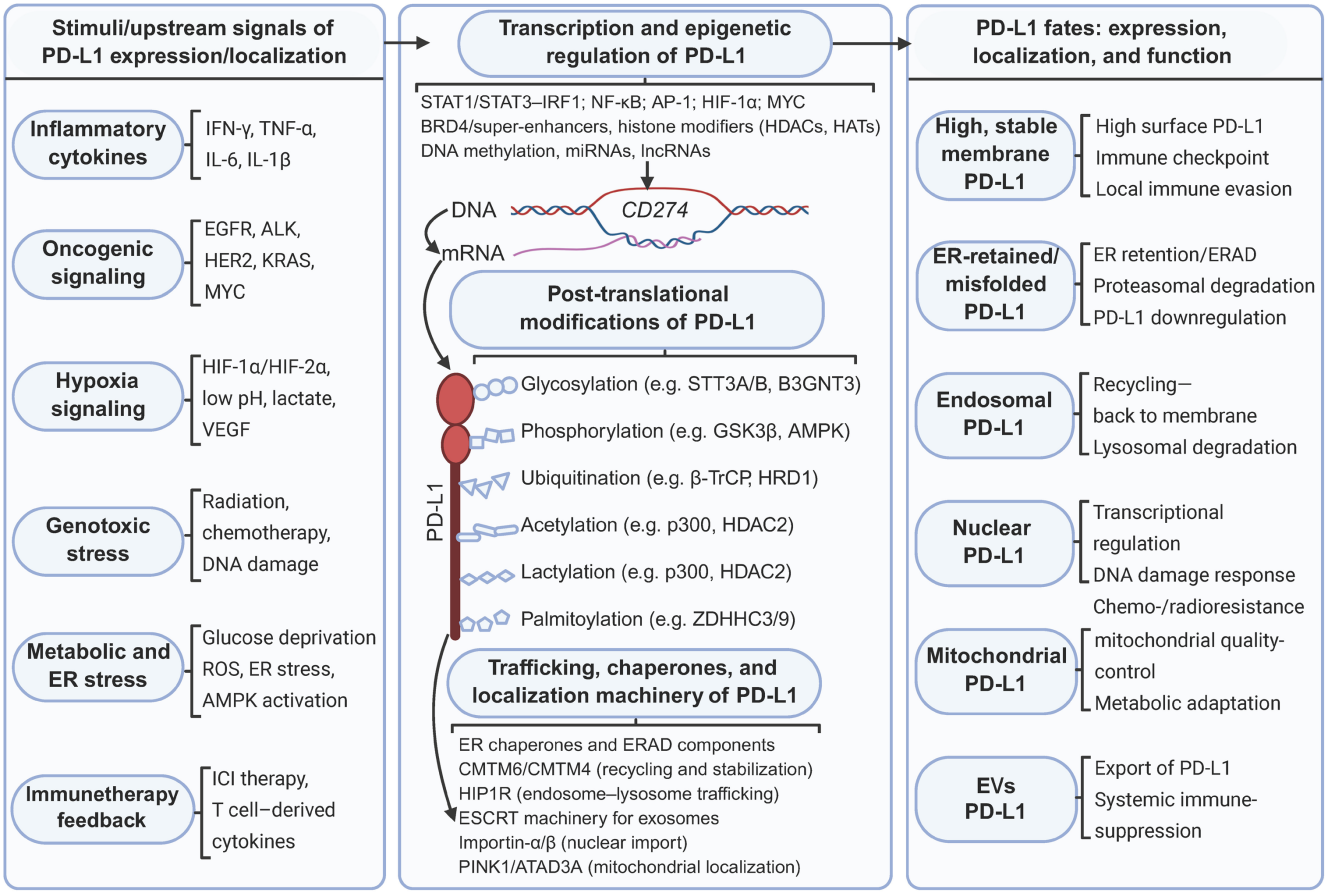
** Regulatory network controlling PD-L1 expression, posttranslational modification and subcellular localization.** Diverse signals in the tumor microenvironment activate distinct pathways that regulate PD-L1 expression. Intracellular PD-L1 is subject to extensive PTMs, which in turn influence its stability and transportation. Chaperones and transport factors coordinate the routing of PD-L1 to different compartments. Created with BioRender.

In summary, a plethora of signals originating from the tumor microenvironment—including inflammatory cytokines, oncogenic signaling cascades, hypoxia, genotoxic stress, and metabolic or ER stress—trigger signaling pathways that converge at the *CD274* gene locus and regulate its protein synthesis. At the transcriptional level, PD-L1 expression is tightly controlled by a repertoire of transcription factors and epigenetic regulators, notably STATs, IRFs, NF-κB, HIFs, and MYC. Following synthesis, PD-L1 undergoes extensive PTM, including glycosylation, phosphorylation, ubiquitination, acetylation, lactylation, and palmitoylation. These PTMs profoundly influence PD-L1 protein stability and its dynamic interactions with intracellular trafficking machinery. A network of molecular chaperones and transport factors distributed across the ER, endosomal-lysosomal system, nuclear import pathways, mitochondrial quality-control machinery, and ESCRT subsequently orchestrates PD-L1 subcellular trafficking. This intricate network directs PD-L1 to distinct destinations: the plasma membrane, recycling endosomes, lysosomes, the nucleus, mitochondria, and EVs. Collectively, these interconnected transcriptional, posttranslational, and trafficking regulatory layers converge to dictate PD-L1 cellular abundance, PTM status, and subcellular localization. In turn, these properties define the dual functional role of PD-L1: as a canonical plasma membrane immune checkpoint that suppresses antitumor immunity and as an intracellular signaling mediator that drives tumor cell-intrinsic survival and therapeutic resistance (Figure 3).

## 5. Biological Functions of Intracellular PD-L1

While membrane-bound PD-L1 suppresses antitumor immunity through T-cell inhibition, intracellular PD-L1 participates in cell-autonomous processes, including transcriptional regulation, the DNA damage response, metabolic reprogramming, and cell survival, that do not require immune cell interactions.

### 5.1 Cytoplasmic Functions

#### 5.1.1 Interaction with Signaling Pathways

Cytoplasmic PD-L1 interacts with multiple oncogenic signaling cascades. The STAT3 pathway is a particularly important nexus—phosphorylated STAT3 physically interacts with PD-L1 to facilitate nuclear translocation, while nuclear PD-L1 subsequently cooperates with STAT3 to regulate the transcription of immune checkpoint and inflammatory genes 98. In hepatocellular carcinoma (HCC), the NUAK1/GSK3β/β-catenin/PD-L1 signaling axis regulates both PD-L1 expression and downstream immunosuppressive effects. Mechanistically, NUAK1 phosphorylates and inactivates GSK-3β, reduces β-catenin degradation, promotes its nuclear translocation, upregulates PD-L1 transcriptionally and promotes immune escape from liver cancer 99. PD-L1 also regulates TGF-β signaling. In hepatic stellate cells, PD-L1 binds and stabilizes TGF-β receptor II (TβRII) at the plasma membrane and accompanies it during endocytosis, ensuring proper SMAD3 activation. PD-L1 deficiency accelerates TβRII sorting to lysosomes, demonstrating a broader role in controlling receptor trafficking beyond its own localization 100. These findings suggest that cytoplasmic PD-L1 interacts with multiple signaling pathways and participates in regulating tumor metabolism and immune escape.

#### 5.1.2 Regulation of Autophagy

PD-L1 can modulate autophagy through the regulation of the PI3K/Akt/mTOR pathway. In pulmonary fibrosis, high expression of PD-L1 inhibits autophagy and promotes disease progression. Intervention with anti-PD-L1 monoclonal antibody restores and enhances autophagic flux by inhibiting the PI3K/Akt/mTOR pathway, thus alleviating pulmonary fibrosis; blocking autophagy, on the other hand, counteracts the antifibrotic effect of anti-PD-L1 antibodies 101. Conversely, PD-L1 itself can undergo autophagic degradation. HIP1R regulates PD-L1 levels through the autophagy-associated lysosomal degradation pathway. It directly binds to PD-L1 and delivers it to lysosomes for degradation via its lysosomal sorting signal, mediated by the AP complex, ALIX and ESCRT-III. Knocking down HIP1R causes PD-L1 accumulation and suppresses T-cell-mediated tumor cytotoxicity 84. These findings suggest that PD-L1 and autophagy have a bidirectional regulatory relationship under different conditions. PD-L1 can regulate the occurrence of autophagy, and autophagy, in turn, controls the expression of PD-L1.

#### 5.1.3 Metabolic Reprogramming

Moreover, intracellular PD-L1 promotes metabolic reprogramming characteristics in cancer cells. In bladder cancer, loss of the nuclear receptor RORC leads to PD-L1 upregulation, which then binds to ITGB6 and activates the FAK/AKT/STAT3 pathway, significantly promoting the Warburg effect and glycolysis in tumor cells and increasing glucose uptake and lactate and ATP production 102. In addition, PD-L1 can directly activate the Akt/mTOR pathway in tumor cells, promoting the translation of glycolytic enzymes and increasing tumor glycolysis and glucose uptake. Blocking PD-L1 with an antibody inhibits Akt/mTOR signaling, reduces glycolytic enzyme levels, weakens tumor glycolytic capacity, and reduces glucose consumption 103. In liver cancer, delactation of PD-L1 at the K189 site leads to its nuclear translocation via vimentin, which in turn upregulates the transcription of the cholesterol synthesis rate-limiting enzyme SQLE through the transcription factor YY1, promoting cholesterol synthesis and accelerating the proliferation of liver cancer cells. Conversely, p300 catalyzes lactation at this site, thereby inhibiting the PD-L1-mediated regulation of cholesterol metabolism 85. This tumor-intrinsic paradigm demonstrates that PD-L1 functions as a signaling adaptor for metabolic and survival pathways independent of immune checkpoint activity.

#### 5.1.4 Regulation of Apoptosis and Survival

Importantly, PD-L1 delivers pro-survival signals to tumor cells independent of PD-1 signaling. By binding to PD-1 on the surface of T cells, PD-L1 transmits antiapoptotic signals to tumor cells through its intracellular domain, enabling tumors to resist CTL-mediated death and Fas- and asteroidin-induced apoptosis, resulting in the formation of a molecular barrier. Moreover, this result does not depend on the inhibitory effect of PD-1 on T cells. Truncation of its intracellular domain can eliminate apoptosis resistance and improve the effect of immunotherapy 104. Furthermore, the intracellular domain of PD-L1 contains the conserved motifs RMLDVEKC and DTSSK, with the former being essential and the latter negatively regulating antiapoptotic signaling. This signaling can antagonize interferon cytotoxicity, inhibit STAT3 phosphorylation and Caspase-7 activation, and promote cancer cell survival. Moreover, mutation of DTSSK in PD-L1 in tumor cells significantly enhances its anti-IFN cytotoxic effect 105. In summary, PD-L1 can directly deliver antiapoptotic signals to tumor cells without relying on PD-1, resulting in the formation of a survival-promoting molecular barrier.

#### 5.1.5 Metastasis and Invasion

Bidirectional feedback occurs between PD-L1 and epithelial‒mesenchymal transition (EMT), a key driver of metastasis. In cancer stem cells, EMT regulates PD-L1 expression through the β-catenin/STT3 axis. Mechanistically, EMT upregulates the N-glycosyltransferase STT3 via β-catenin/TCF4 transcription. STT3 mediates the N-glycosylation of PD-L1 to stabilize the protein and increase its expression, leading to PD-L1 accumulation in cancer stem cells and promoting immune escape. Chemotherapy with etoposide can reverse EMT, inhibit this pathway, and downregulate PD-L1 expression 72. In EGFR-mutant NSCLC cells, PD-L1 activates the TGF-β/Smad pathway by upregulating Smad3 phosphorylation, inducing EMT and enhancing migration and invasion capabilities, leading to primary resistance of tumor cells to gefitinib. Silencing PD-L1, on the other hand, inhibits EMT and metastasis 106. In summary, the mutual regulation of PD-L1 and EMT represents an important mechanism that promotes tumor metastasis, invasion, and immune escape. Intervening in this association may effectively curb tumor metastasis.

### 5.2 Nuclear Functions

#### 5.2.1 Transcriptional Regulation

Nuclear PD-L1 possesses DNA-binding capabilities and functions as a transcriptional regulator, mediating the transcription of multiple immune response-related genes involved in IFN-γ-mediated signaling, NF-κB signaling, and antigen processing/presentation by MHC-I. Notably, the expression of immune checkpoint genes, including PD-L2, VISTA, and B7-H3, is downregulated by CD274 depletion, suggesting that nuclear PD-L1 contributes to resistance mechanisms through the coordinated expression of multiple checkpoint molecules 10. In uveal melanoma, nuclear PD-L1 is deacetylated by HDAC2 and enters the nucleus, where it recruits p-STAT3 to bind to the EGR1 promoter, where it is transcribed and activates EGR1 and upregulates VEGFA, thus promoting tumor angiogenesis 107 In ovarian cancer, nuclear PD-L1 functions as a transcriptional coactivator by being recruited to specific gene promoters (IL-8, Bcl3, and STAT1), promoting histone acetylation at upstream regulatory regions and facilitating RNA polymerase II recruitment 108. Therefore, nuclear PD-L1, with its transcriptional regulatory capacity, directly regulates the expression of oncogenes and indirectly regulates the immune microenvironment by affecting the activation of immune-related genes, making it an important factor influencing the malignant progression of tumors.

#### 5.2.2 DNA Damage Response and Repair

A landmark discovery revealed that intracellular PD-L1 acts as an RNA-binding protein that regulates the mRNA stability of DNA damage response genes. The K270-T279 sequence in the cytoplasmic domain of PD-L1 binds target mRNAs including NBS1 and BRCA1, protecting them from degradation by competing with the RNA exosome complex. PD-L1 knockdown shortened the half-lives of NBS1 and BRCA1 mRNA, increasing cellular sensitivity to DNA-damaging agents 109. Nuclear PD-L1 also directly participates in DNA double-strand break repair. In an NSCLC cell model, ionizing radiation induces PD-L1 deglycosylation, triggering nuclear translocation where PD-L1 interacts with Ku80 through its IgC domain. This interaction enhances Ku/DNA binding and promotes Ku interactions with DNA-PKcs and XRCC4, facilitating NHEJ 74. Conversely, tumor-intrinsic PD-L1 promotes BRCA1-mediated homologous recombination by co-immunoprecipitating with BARD1 and increasing BRCA1 nuclear accumulation 110. This mechanism has been validated in human bladder (t24) and breast cancer cells (4T1, MDA-MB-231) and in mouse melanoma cells (B16-F10). This controversy may stem from differences in the genetic backgrounds, PD-L1 subcellular localization ratios, and experimental detection systems of the cell lines, suggesting that nuclear PD-L1 regulation of the DSB repair pathway is highly context dependent.

#### 5.2.3 Cell Cycle Control

Nuclear PD-L1 also regulates cell cycle progression and proliferation through multiple mechanisms. In BRAF-mutated CRC, p-ERK mediates PD-L1 nuclear translocation, and nuclear PD-L1 binds to and inhibits THRAP3, relieves its inhibition of BUB1 and upregulates BUB1 expression, accelerates the cell cycle G1/S process, and promotes tumor cell proliferation. 38. During triple-negative breast cancer cell division, PD-L1 exists as a subunit of the adhesin complex, compensating for the loss of Sororin and competing with WAPL for binding to PDS5B to ensure proper sister chromatid cohesion and segregation. Depleting PD-L1 leads to multinuclear cells and suppresses proliferation both *in vitro* and *in vivo* in immunodeficient mice 111. These findings demonstrate that nuclear PD-L1 directly accelerates tumor proliferation by driving cycle transitions and ensuring chromosome stability, highlighting its key role as an intrinsic oncogenic driver.

#### 5.2.4 Stem Cell Maintenance

Nuclear PD-L1 participates in maintaining tumor cell stemness through distinct mechanisms. In cancer stem cells, EMT promotes N-glycosylation and protein stabilization of PD-L1 through the β-catenin/STT3 axis, leading to high PD-L1 expression and immune escape. This regulation is transcription independent and mainly maintains the stem cell-associated immune resistance phenotype through glycosylation 72. In breast cancer, nuclear PD-L1 is a key molecule that regulates stemness, and its expression is regulated by BRD4 transcription. Nuclear PD-L1 positively regulates RelB, which forms a complex with p65 and activates IL-6 transcription, in turn maintaining the stemness of cancer cells 112. In this context, nuclear PD-L1 functions through indirect transcriptional regulation. The connection between nuclear PD-L1 and stemness suggests that targeting intracellular PD-L1 may reduce both immune evasion and tumor-initiating capacity.

#### 5.2.5 Chemoresistance and Radioresistance

Mediating resistance to therapy is an important manifestation of the tumor-promoting ability of nuclear PD-L1. Following doxorubicin treatment, PD-L1 translocates from the membrane to the nucleus concomitant with phosphorylated Akt, promoting drug resistance 97. The RNA-binding function of PD-L1 protects NBS1 and BRCA1 mRNAs from degradation, increasing resistance to DNA-damaging chemotherapy and radiation 109. Anti-PD-L1 antibody, which promotes PD-L1 degradation, significantly enhanced tumor sensitivity to radiation and cisplatin in both immunodeficient and immunocompetent mice. In addition, the role of nuclear PD-L1 in enhancing NHEJ through Ku80 interaction directly contributes to radioresistance 74. It is evident that nuclear PD-L1 is a key factor that mediates tumor resistance to radiotherapy and chemotherapy. It can reduce the sensitivity of tumors to chemotherapy and radiotherapy through multiple mechanisms, thereby weakening the therapeutic effect. Preclinical observations have shown that targeted promotion of its degradation can significantly increase sensitivity to radiotherapy and chemotherapy, providing a potential approach for reversing resistance to tumor treatment.

#### 5.2.6 Context-dependent Antitumor Effects

Paradoxically, some studies have also reported antitumor effects of nuclear PD-L1. The nuclear translocation of PD-L1 is mediated by alpha-lipoic acid-activated p-AMPKα. By cophosphorylating histone macroH2A1 at the S146 site with p-AMPKα, it epigenetically activates cellular senescence, JAK/STAT, and Hippo signaling pathways; upregulates the expression of MHC-I and IFNGR in tumor cells to enhance CD8^+^ T-cell-mediated antitumor immunity; significantly inhibits the occurrence and progression of HCC; and can synergize with PD-1 monoclonal antibodies to effectively overcome resistance to immune checkpoint blockade therapy in HCC, CRC, and pancreatic cancer 113. In breast cancer, hypoxia induces nuclear PD-L1 to bind to phosphorylated Stat3, transcribing and activating GSDMC, which is then cleaved by TNFα/caspase-8, leading to pyroptosis. Although nuclear PD-L1-mediated chronic tumor necrosis generally promotes tumor progression, the pyroptosis pathway it regulates can be effectively activated by antibiotic chemotherapy drugs (such as doxorubicin, epirubicin, and actinomycin D), enhancing antitumor immunity through immunogenic cell death, suggesting that targeting this pathway can improve the immunotherapeutic effect of chemotherapy 92. In conclusion, nuclear PD-L1 has context-dependent tumor regulatory effects. In specific tumor types and in response to specific microenvironmental stimuli, it can also activate tumor suppressor cell programs, enhance antitumor immunity, and synergize with immunotherapy to improve the immune-killing effect of chemotherapy. On this basis, a more precise, individualized treatment strategy may be considered.

### 5.3 Functions in Mitochondria

Mitochondrial PD-L1 determines the response to chemotherapy and immunotherapy in triple-negative breast cancer, and the abundance of mitochondrial PD-L1 is positively correlated with treatment efficacy. The ATAD3A-PINK1 axis regulates PD-L1 mitochondrial transport and autophagic degradation. The disruption of this axis with paclitaxel alters mitochondrial PD-L1 levels, with consequences for tumor cell survival and therapeutic responses 55. In HCC, IFN-γ can induce the mitochondrial translocation of PD-L1, which in turn activates Drp1 to mediate mitochondrial division, reprogram glycolysis, and upregulate GPX4 expression to inhibit sorafenib-induced ferroptosis, thus increasing the stemness of liver cancer stem cells. Targeted intervention of mitochondrial PD-L1 can reverse the above effects, sensitizing cells to sorafenib and inhibiting liver cancer stemness 114. In summary, PD-L1 transport to mitochondria can serve as a biomarker for treatment response, but conversely, it can enhance tumor stemness and drug resistance by remodeling mitochondrial activity. More cell-specific evidence is needed to understand the relationship between mitochondrial PD-L1 expression and tumor treatment.

### 5.4 Functions in Extracellular Vesicles

EV-PD-L1 mediates intercellular communication with profound immunological consequences. Unlike membrane PD-L1, which acts locally at the tumor-T-cell interface, EV-PD-L1 suppresses immune responses systemically and circulates throughout the body to affect T cells in the lymph nodes, bloodstream, and surrounding tissues. EV-PD-L1 is more potent than membrane PD-L1 for inducing T-cell dysfunction, which is attributed to greater stability through MHC-I association 57. Tumor-derived EVs carrying PD-L1 are likely to create favorable immunosuppressive microenvironments at distant sites before metastatic cancer cells arrive, facilitating the establishment of metastasis 115. In summary, EV-PD-L1 overcomes local limitations to achieve systemic immunosuppression, resulting in stronger efficacy and a more stable structure, and can remotely preprogram the premetastatic immune exemption environment. This mechanism reveals that the invasion strategy of tumors actively reshapes the global immune landscape through EVs and is a factor that should be emphasized in future tumor immunotherapy.

**Figure 4:**
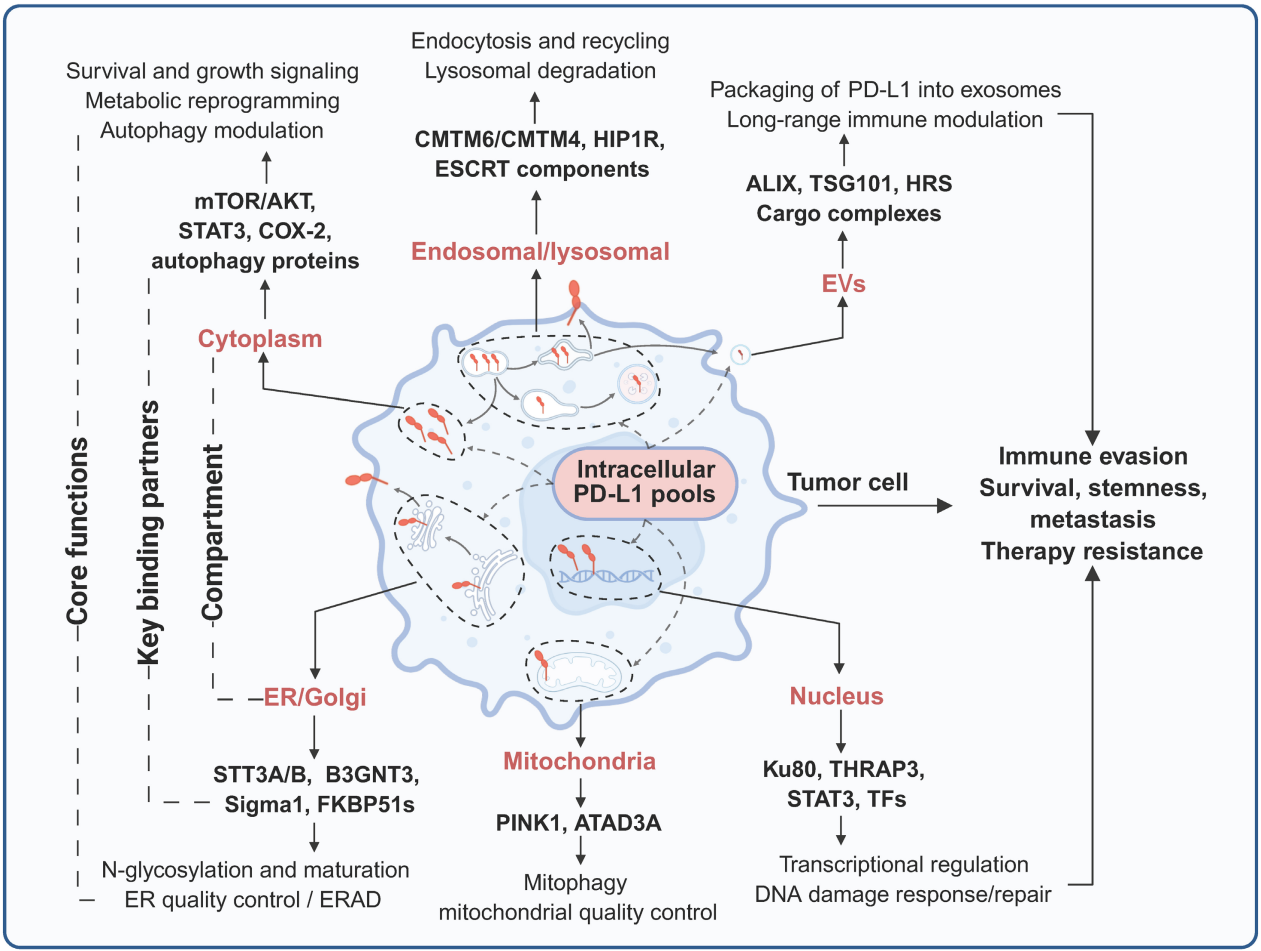
** Compartmentalized interactome and functions of intracellular PD-L1.** PD-L1 resides in multiple subcellular compartments, where it engages distinct sets of binding partners to execute diverse functions. Created with BioRender.

In conclusion, PD-L1 is localized to multiple distinct subcellular compartments, where it engages compartment-specific interactomes to execute context-dependent biological functions. In the cytoplasm, PD-L1 engages with key components of cell survival, metabolic signaling, and autophagy regulatory networks, thereby regulating tumor cell proliferation, stress adaptation, and metabolic reprogramming. Nucleus-localized PD-L1 interacts with transcriptional coregulators and DNA damage response machinery to modulate global gene expression programs and DNA repair fidelity in a context-dependent manner in order to regulate tumor progression. In the ER and Golgi apparatus, PD-L1 associates with molecular chaperones and glycosyltransferases to undergo glycosylation and protein quality control—processes that dictate its proper folding, maturation, and proteolytic stability. Within the endosomal-lysosomal system, PD-L1 forms complexes with vesicular trafficking proteins that fine-tune the balance between its recycling to the plasma membrane and its lysosomal degradation, thereby precisely controlling its cell surface abundance. Mitochondrial PD-L1 participates in the regulation of mitophagy and mitochondrial bioenergetic adaptation to cellular stress. Additionally, PD-L1 is packaged into EVs, which disseminate systemic immunosuppressive signals and precondition distant metastatic niches. Collectively, these spatially segregated, compartment-specific interactomes integrate extracellular environmental signals and intracellular oncogenic cues to coordinately regulate tumor immune evasion, malignant progression, and therapeutic resistance (Figure 4, Table 2).

## 6. Clinical Significance of Intracellular PD-L1

### 6.1 Prognostic Value

Emerging evidence suggests that the subcellular distribution of PD-L1 provides prognostic information beyond total expression. In uveal melanoma, high expression of nuclear PD-L1 is significantly associated with poor prognosis and early tumor recurrence. Nuclear PD-L1 recruits p-STAT3 to activate the EGR1/VEGFA pathway, which promotes tumor angiogenesis. High expression of key molecules in this pathway also indicates poor prognosis 107. Delactylation of PD-L1 K189 promotes its nuclear translocation, upregulating the cholesterol synthesis rate-limiting enzyme SQLE via the transcription factor YY1, thus accelerating hepatocellular carcinoma growth. Clinically, PD-L1 K189 lactylation levels are negatively correlated with HCC pathological grade, and low PD-L1 expression is an independent predictor of poor prognosis in patients with HCC 85. Another retrospective clinical study revealed that nuclear PD-L1 is expressed in circulating tumor cells that are positive for vimentin. The mere number of circulating tumor cells is not significantly correlated with the prognosis of patients with colorectal or prostate cancer. However, nuclear PD-L1 positivity indicates a shorter survival period and can serve as a potential prognostic marker for both types of metastatic cancers 116. In BRAF-mutated CRC, 72.7% of tumors demonstrate significant nuclear PD-L1 staining, which is correlated with tumor invasion and poor outcomes 38. Moreover, EV-PD-L1 serves as a particularly promising prognostic biomarker; high pretreatment levels predict poor prognosis in patients with metastatic melanoma, whereas high posttreatment increases indicate adaptive responses to T-cell reinvigoration 117. In summary, clinical investigations in various tumors have confirmed that the abundance of PD-L1 in subcellular locations (especially nuclear PD-L1) can provide prognostic information beyond the total expression level. Moreover, nuclear PD-L1 positivity in circulating tumor cells and the level of Ev-PD-L1 are closely related to poor patient prognosis. This fully highlights the clinical value of intracellular PD-L1 as a new prognostic marker, which is expected to overcome the limitations of traditional total PD-L1 expression and provide a key basis for precise risk stratification and individualized treatment decisions for tumor patients.

### 6.2 Predictive Biomarkers for Immunotherapy

Current research on PD-L1 as a predictive biomarker for the efficacy of tumor immunotherapy focuses mainly on EV-PD-L1. Serum EV-PD-L1 levels in patients with advanced pancreatic cancer are correlated with PD-L1 positivity in tumor tissue, and metastatic patients have higher EV-PD-L1 levels, which are associated with shorter overall survival after PD-1/PD-L1 monotherapy, suggesting that serum EV-PD-L1can serve as a predictive biomarker for immunotherapy efficacy 118. In patients with metastatic melanoma, circulating EV-PD-L1 levels are positively correlated with IFN-γ levels and vary during anti-PD-1 therapy. In the early stages of treatment, the magnitude of the increase in circulating EV-PD-L1, as an indicator of the adaptive response of tumor cells to T-cell reactivation, can distinguish clinical responders from nonresponders. However, high pretreatment EV-PD-L1 levels suggest poor anti-PD-1 efficacy 119. Another prospective clinical study on melanoma reported that the positivity rate of EV-PD-L1 in the peripheral blood of patients is greater than that in tumor tissue. Elevated EV-PD-L1 levels after treatment indicate disease progression, can distinguish between treatment responders and patients with progression, and can predict progression-free survival and overall survival, allowing the dynamic monitoring of treatment efficacy 120. Recent studies have reported that a high-affinity platform based on dendrimer‒peptide conjugates can efficiently capture EV-PD-L1. Clinical validation based on this technology has shown that compared with tissue PD-L1 scores, EV-PD-L1 levels are better predictors of lung cancer immunotherapy response, and high EV-PD-L1 levels before immunotherapy indicate poor efficacy and shorter overall survival 121. Taken together, the results of multiple clinical studies have confirmed that circulating EV-PD-L1 levels are closely associated with immunotherapy response and survival prognosis in patients with pancreatic cancer, melanoma, lung cancer and other tumors; may be used for dynamic monitoring of therapeutic efficacy; and may have better predictive value than tissue PD-L1 expression scores do. A high-efficiency EV-PD-L1 capture technology has also been developed and clinically validated, further demonstrating its promise as a biomarker.

### 6.3 Immunotherapy Target

Given the extensive distribution of PD-L1 in intracellular compartments and its diverse nonimmune regulatory functions, targeting intracellular PD-L1 may represent a promising therapeutic strategy for tumors. Cytoplasmic PD-L1, an RNA-binding protein, competes with the components of RNA exosomes for binding and stabilizing the mRNA of DNA damage repair genes, enhancing DNA repair in tumor cells to mediate resistance to radiotherapy and chemotherapy. By blocking the binding of PD-L1 to CMTM6 using an H1A antibody in animal models of melanoma and breast cancer, the lysosomal degradation of PD-L1 can be promoted, inhibiting the DNA damage response, achieving radiotherapy and chemotherapy sensitization, and possessing dual potential for immune regulation and therapeutic sensitization 109. Radiotherapy induces the deglycosylation of PD-L1 in lung cancer H460 cells, which are then transported into the nucleus via CMTM6. Nuclear PD-L1 binds to Ku, enhancing the NHEJ pathway for DNA double-strand break repair and mediating tumor radioresistance. By targeting nuclear PD-L1 through blocking its desulfation, nuclear transport, or binding to Ku, it may reverse radioresistance and relieve immune suppression 74. However, in some cases, such as HCC, the activation of PD-L1 nuclear translocation may be helpful for immunoblockade therapy 113. EV-PD-L1 binds to PD-1 on distant CD8+ T cells, inhibiting their proliferation, death, and cytokine secretion. This is an important mechanism through which Ev-PD-L1 mediates tumor immune escape. In NSCLC cells, LAMTOR1 can bind to HRS to promote PD-L1 lysosomal degradation, thereby reducing exosomal PD-L1 expression. The combination of LAMTOR1-targeting peptides and anti-PD-1 immunotherapy in an NSCLC mouse model can significantly restore T-cell function and enhance antitumor efficacy 122. Overall, targeting of intracellular PD-L1 has dual anti-tumor potential through immune regulation and therapeutic sensitization by intervening in tumor radiotherapy and chemotherapy resistance as well as immune escape mediated by cytoplasmic, nuclear and EV-PD-L1; however, the relevant evidence is derived only from preclinical cellular and animal studies, indicating that clinical trial exploration is urgently needed for its clinical application in tumor therapy.

## 7. Therapeutic Implications and Strategies

### 7.1 Limitations of Current Immunotherapies

Existing FDA-approved anti-PD-L1/PD-1 antibodies function exclusively by blocking extracellular PD-1/PD-L1 interactions at the cell surface. These therapeutic antibodies cannot penetrate cell membranes to access intracellular PD-L1 pools, leaving a substantial reservoir of functional PD-L1 untouched. Multiple clinical scenarios illustrate the inadequacy of membrane-targeted approaches. BRAF-mutated CRC frequently exhibit high PD-L1 expression yet respond poorly to checkpoint blockade; corresponding preclinical *in vitro* studies have confirmed that this paradox may be caused by predominant nuclear PD-L1 localization, which drives proliferation through THRAP3-BUB1 signaling independent of PD-1 engagement 38. Similarly, treatment-induced PD-L1 upregulation following chemotherapy or radiation activates nuclear translocation and DNA repair functions that counteract therapeutic efficacy 123. This fundamental pharmacological limitation means that the immune-independent functions of intracellular PD-L1 remain unaddressed. Critical preclinical evidence reveals that intracellular PD-L1 mediates resistance to therapy that is inaccessible to antibody-based blockade. The RNA-binding activity of PD-L1 prevents the degradation of NBS1 and BRCA1 mRNA, thereby conferring resistance to DNA-damaging agents 109. The H1A antibody, which disrupts the PD-L1-CMTM6 interaction and promotes PD-L1 degradation, markedly sensitizes tumors to radiotherapy and cisplatin in a breast and melanoma tumor mouse model—an effect not recapitulated by durvalumab 109. Likewise, conventional anti-PD-L1 antibodies fail to increase sensitivity to PARP inhibitors in subcutaneous transplantation tumor models of melanoma and breast cancer, whereas the genetic depletion of PD-L1 triggers a significant antitumor effect, indicating that intracellular PD-L1 pools are involved in therapy resistance 110. In summary, intervention strategies targeting cell surface PD-L1 failed to effectively target and interfere with the immune-independent functions of intracellular PD-L1. This may partially explain the clinical paradoxes, such as the poor response of tumors with high PD-L1 expression to immune checkpoint blockade and the counterproductive effects of radiotherapy- and chemotherapy-induced upregulation of PD-L1 on therapeutic outcome. This highlights the urgent need to develop new therapeutic strategies targeting intracellular PD-L1 to overcome the existing limitations of immunotherapy and solve the problem of treatment resistance that antibodies cannot overcome.

### 7.2 Novel Targeting Approaches

#### 7.2.1 Accessing Intracellular PD-L1

A prerequisite for targeting intracellular PD-L1 for intervention is that the drug can enter the cell. Cell-penetrating peptides conjugated to PD-L1-targeting moieties offer potential access to intracellular compartments. The PD-PALM peptide, designed as a cell-penetrating peptide that blocks PD-L1 palmitoylation at C272, promotes its lysosomal degradation *in vitro* 86. On the basis of lysosome-dependent PD-L1 protein degradation mediated by HIP1R, a chimeric PD-LYSO peptide that combines PD-L1 binding sequences with lysosomal sorting signals from HIP1R was constructed and validated to effectively target PD-L1 for lysosomal degradation in cancer cells 84. Small molecules offer inherent advantages for accessing intracellular targets. Similarly, the small molecule MS1-96 directly binds to PD-L1 and promotes HIP1R-dependent lysosomal PD-L1 degradation, pharmacologically redirecting intracellular PD-L1 from recycling endosomes to lysosomes. Furthermore, the ability of MS1-96 to induce the degradation of PD-L1 can suppress the growth of CRC xenograft tumors by activating tumor-infiltrating CD8^+^ T cells 124. Another small-molecule lead compound, CB31, demonstrates outstanding potency in blocking PD-1/PD-L1 interactions with favorable oral pharmacokinetics. Importantly, CB31 can also induce PD-L1 internalization, alter its glycosylation pattern, and promote its degradation. In a 3D tumor spheroid model, CB31 intervention significantly inhibited tumor growth, killing of tumor cells, and infiltration of CD8^+^ T cells 125. Taken together, these findings suggest that cell-penetrating peptides and small molecules represent two promising delivery and intracellular PD-L1 targeting strategies. These innovative strategies successfully overcome the core limitations of traditional antibodies that cannot penetrate the cell membrane, providing a preclinical basis for the development of a new generation of tumor immunotherapy drugs. In addition, the use of single-chain variable fragments (scFvs) and nanobodies, which can be designed to be specifically expressed in certain cellular substructures, is also emerging as a type of intracellular PD-L1-targeting technology that is worth exploring.

#### 7.2.2 Targeted Protein Degradation

Directly targeting and degrading intracellular PD-L1, which has a tumor-promoting effect, is an obvious antitumor strategy. Proteolysis-targeting chimeras (PROTAC) technology enables proteasome-dependent protein degradation through the recruitment of E3 ubiquitin ligases to proteins of interest. BMS-37-C3, developed by combining PD-L1 small-molecule inhibitors with PROTAC technology, results in dose- and time-dependent PD-L1 reduction in A375 and B16-F10 cell lines 126. Moreover, lysosome-targeting chimera (LYTAC) technology specifically targets proteins for lysosomal degradation. A recently reported DNA aptamer covalent LYTAC-DBCO chimera, with one end binding to the lysosomal transport receptor CI-M6PR and the other end covalently coupled to PD-L1 via DBCO, was constructed to mediate the lysosomal degradation of PD-L1. Observations in melanoma animal models and *in vitro* models revealed that this LYTAC could significantly induce lysosome-dependent PD-L1 degradation, induce tumor immunogenic apoptosis, and enhance antitumor immunity, and its efficacy was superior to that of PD-L1 antibodies, with lower immune-related damage 127. In addition, antibody-based targeting chimeras (AbTACs) use bispecific antibodies that can bind to PD-L1 and recruit the intracellular E3 ubiquitin ligase RNF43 to mediate the lysosomal degradation of PD-L1, thereby overcoming the limitations of traditional PROTACs in targeting membrane proteins 128. Furthermore, dihydropyridine derivatives have been reported to be optimized through structural-activity relationships to obtain compound F4, which degrades PD-L1 in a lysosome-dependent manner and can enhance the tumor-killing effect mediated by T cells, providing a new strategy for small-molecule immunotherapy 129. Targeted protein degradation technology provides an efficient strategy for targeting intracellular PD-L1. Various techniques, such as PROTAC, LYTAC, AbTAC and small-molecule degraders, can specifically induce PD-L1 degradation through the proteasome or lysosome pathways, resulting in antitumor immune effects superior to those of traditional PD-L1 antibodies. This fully demonstrates the clinical transformation potential of these technologies in overcoming the limitations of existing immunotherapy.

#### 7.2.3 Blocking Intracellular Functions

Restricting the intracellular transport of PD-L1 is the key step that hinders its function. VPS18 and VPS11 regulate PD-L1 through trans-Golgi network recycling; the small-molecule inhibitor RDN, which targets VPS18, impairs PD-L1 trafficking and protein stability and enhances antitumor immunity when combined with anti-CTLA-4 in metastatic melanoma tumors and drug-resistant prostate cancer in mice 43. CMTM6 disruption redirects PD-L1 from recycling to degradation; the H1A antibody abolishes CMTM6 binding, promoting PD-L1 lysosomal degradation, which sensitizes cancer to DNA-damaging therapy in a breast and melanoma cancer mouse model 109. HDAC2 inhibitors maintain K263 acetylation, blocking PD-L1-HIP1R interaction and subsequent nuclear translocation; the combination of an HDAC2 inhibitor with anti-PD-1 substantially slows tumor growth and increases survival in a mouse colon cancer model 10. This preclinical evidence suggests that targeting intracellular PD-L1-interacting proteins, trafficking, and PTMs can block its nonimmune regulatory functions, thereby producing direct antitumor effects or reducing resistance to therapy. In conjunction with these findings, many potential therapeutic targets focused on PD-L1 signaling have been reported. Nuclear PD-L1 functions in tumor progression through specific protein interactions with context-dependent characteristics, which may be therapeutically targeted. The PD-L1-THRAP3 interaction drives BUB1 upregulation and cell cycle progression in BRAF-mutated CRC 38. Nuclear PD-L1-Ku80 interaction enhances NHEJ-mediated DNA repair; peptides or small molecules that disrupt this interaction could sensitize tumors to radiation 74. In addition, modulating PTMs represents another intervention strategy. Targeting the glycosylation machinery—including STT3, B3GNT3, Sigma1, and FKBP51 s—may disrupt PD-L1 maturation and stability 70. GSK3β activators promote the phosphorylation-dependent degradation of nonglycosylated PD-L1 71. CSN5 inhibitors prevent deubiquitination, thereby promoting PD-L1 degradation 81. ZDHHC3 inhibitors block PD-L1 C272 palmitoylation, thereby promoting autophagic degradation 86. In summary, in addition to directly targeting and degrading intracellular PD-L1, targeting PD-L1-interacting proteins and regulating the maturation, stability, and subcellular localization of PD-L1 through various PMTs, such as glycosylation, phosphorylation, ubiquitination, and palmitoylation, constitute a highly promising and diversified system of therapeutic targeting strategies in the PD-L1 signaling pathway.

#### 7.2.4 Enhancing Membrane Presentation

Interestingly, strategies that enhance surface PD-L1 expression can increase antitumor efficacy in specific contexts. While this might seem counterintuitive, combining such approaches with checkpoint blockade can enhance target engagement. Dynamin inhibitors such as prochlorperazine decrease PD-L1 internalization, increasing surface expression and enhancing avelumab efficacy through improved NK cell-mediated antibody-dependent cellular cytotoxicity (ADCC) in colon cancer models 90. CDK4/6 inhibitors, which paradoxically increase PD-L1 expression by disrupting SPOP-mediated degradation, prime tumors for checkpoint blockade; moreover, combining palbociclib with anti-PD-1 made immunotherapy more effective in a mouse model of CRC 77. PAI-1 transports membrane PD-L1 to lysosomes for degradation via clathrin-dependent endocytosis mediated by Prolow-density lipoprotein receptor-related protein 1 (LRP1), resulting in low membrane expression of PD-L1. Pharmacological inhibition of PAI-1 by tiplaxtinin can stabilize tumor cell membrane PD-L1 and significantly increase the antitumor efficacy of anti-PD-L1 monoclonal antibodies in a mouse model of melanoma 130. Therefore, under certain conditions, a lack of membrane PD-L1 expression in tumor cells is just as detrimental to the efficacy of ICI therapy as excessive expression of membrane PD-L1. Precisely regulating membrane PD-L1 expression to an appropriate level, rather than simply maintaining or downregulating it, may be a promising direction for optimizing immunotherapy.

#### 7.2.5 Combination Strategies

**Figure 5:**
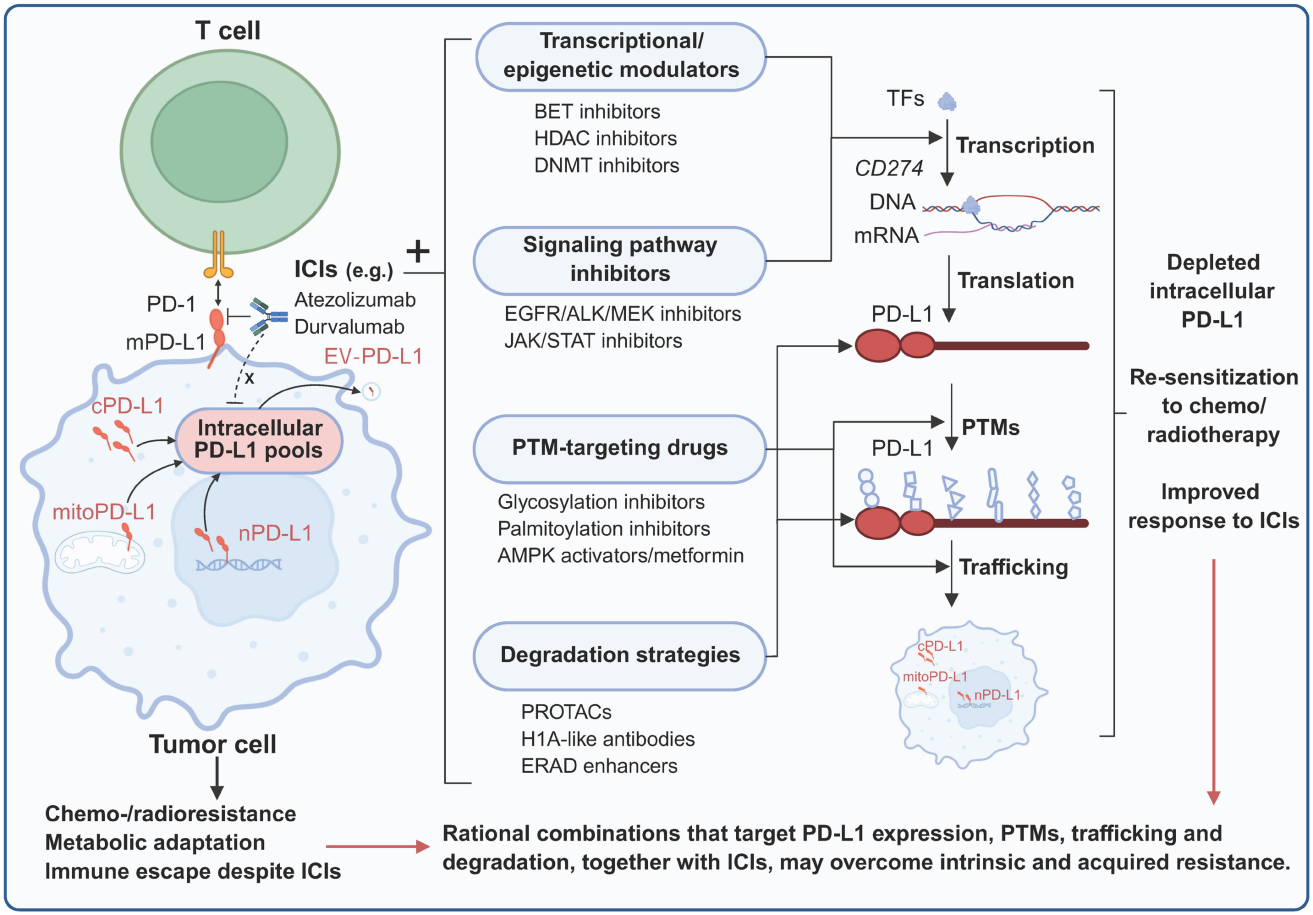
** Clinical implications of intracellular PD-L1.** Intracellular PD-L1 pools represent promising targets for tumor therapy, which can be modulated at multiple levels and combined with existing ICIs to improve response rates. Created with BioRender.

Combining intracellular PD-L1 targeting with ICI therapy can result in synergistic effects. Metformin combined with anti-CTLA4 shows synergistic antitumor activity in a mouse breast tumor model through AMPK-mediated PD-L1 degradation 52. In addition, targeting intracellular PD-L1 can overcome treatment-induced resistance. The BRD4 inhibitor JQ1 suppresses chemoradiation-induced PD-L1 transcription; triple combination therapy (JQ1/chemoradiotherapy/anti-PD-1) results in complete tumor regression in NSCLC models 123. Aucubin, a natural compound that suppresses PD-L1 through the inhibition of Akt/β-catenin activity, blocks cisplatin-induced PD-L1 upregulation and enhances the efficacy of combined treatment in an HCC mouse model 131 Furthermore, the activation of compensatory immunosuppressive pathways that may result from PD-L1 loss can also be overcome through combined strategies. In immune-cold Triple negative breast cancer (TNBC), PD-L1 is expressed at low levels, while another immune checkpoint, B7-H4, exhibits significantly increased expression (similar to the degradation of targeted PD-L1), hindering the response to anti-PD-L1 therapy. In this context, triple therapy simultaneously targets B7-H4 and PD-L1 and activates ICD, resulting in over 90% tumor growth inhibition in preclinical models, which offers promising clinical translation prospects 132. In summary, strategies targeting intracellular PD-L1 can synergistically enhance the effects of immune checkpoint antibodies, radiotherapy, or chemotherapy by complementing their mechanisms of action. By blocking treatment-induced PD-L1 upregulation and drug resistance through multiple pathways, combination strategies can significantly enhance antitumor immune responses and achieve precise tumor inhibition (Figure 5).

## 8. Future Directions

The current understanding of intracellular PD-L1 is largely based on population-level analyses that mask cellular heterogeneity. Single-cell RNA sequencing combined with spatial proteomics will enable the simultaneous evaluation of PD-L1 mRNA expression, protein abundance, and subcellular localization in individual tumor microenvironment cells, helping identify tumor subpopulation-specific PD-L1 functions and spatial distributions. Live-cell imaging with fluorescently tagged PD-L1 and photoactivatable/photoconvertible variants will clarify real-time PD-L1 trafficking kinetics, while super resolution microscopy will visualize PD-L1-trafficking machinery interactions at high resolution. The cryo-electron microscopy-based structural analysis of intracellular PD-L1 complexes, along with investigations of the effects of PTMs on PD-L1 conformation, will facilitate the rational design of targeted agents.

Proximity labeling coupled with mass spectrometry will be used to systematically map PD-L1 interactomes in different subcellular compartments, especially cell-cycle-dependent binding partners, to elucidate unrecognized functions and regulatory mechanisms. Systems biology approaches integrating CRISPR screening, proteomics, and computational modeling will be used to characterize the complete regulatory network governing the subcellular distribution of PD-L1 and identify optimal therapeutic intervention nodes. Systematic comparisons of intracellular PD-L1 functions across cancer types, molecular subtypes, and microenvironmental conditions will elucidate context-dependent therapeutic vulnerabilities. Understanding how microenvironmental stimuli and therapeutic interventions regulate the balance between the immune-dependent and immune-independent functions of PD-L1 is critical for rational therapeutic design.

Knock-in mice with localization-specific PD-L1 variants and conditional models will dissect compartment-specific PD-L1 functions *in vivo* and their effects on therapeutic responses during tumor progression. Patient-derived organoids, especially when cocultured with autologous immune cells, will enable clinically relevant evaluation of intracellular PD-L1 targeting strategies and guide patient selection. Prospective studies correlating subcellular PD-L1 distribution with treatment outcomes are urgently needed, along with the development and validation of standardized assessment methods for subcellular PD-L1 and its modifications. The liquid biopsy-based detection of circulating EV-PD-L1 requires clinical validation to enable longitudinal monitoring.

## 9. Conclusions

The discovery of intracellular PD-L1 has revolutionized the mechanistic understanding of this immune checkpoint protein, shifting the view of PD-L1 from a strictly membrane-bound ligand to a multifunctional regulator distributed in the nucleus, cytoplasm, organelles and EVs. These subcellular PD-L1 pools perform critical biological functions that are independent of canonical PD-1-mediated immune suppression: They modulate transcription, RNA stability, DNA repair, chemoradiotherapy resistance and systemic immunosuppression, and they are tightly regulated by PTMs, subcellular trafficking and microenvironmental stimuli. Notably, anti-PD-L1 antibodies fail to target intracellular PD-L1, leaving a functionally critical pool unexploited, which may partially account for clinical resistance to checkpoint blockade. Harnessing the therapeutic potential of intracellular PD-L1 requires advanced detection strategies, subcellular localization-based biomarkers and rational clinical trials. Integrating the roles of intracellular PD-L1 into precision immunotherapy is a pivotal next step in cancer treatment.

## Funding

This work is supported by the National Natural Science Foundation of China (no. 82504382).

## Figures and Tables

**Table 1:** Post-translational modifications of PD-L1 and their effects on stability, localization and function

Type of modification	Key enzymes	Upstream stimuli/pathways	Effects on PD-L1 protein	Representative functional consequences	References
Glycosylation	STT3A, B3GNT3; ERAD components	IFN-γ-JAK1-STAT1/IRF1 signaling; oncogenic RTKs; inflammatory and hypoxic	Promotes proper folding and maturation; stabilizes PD-L1 and prolongs its half-life; enhances PD-1 binding; sterically blocks GSK3β access to its phosphorylation motif and limits β-TrCP recruitment; may mask epitopes in IHC	Sustained high surface PD-L1 levels and immune evasion; resistance to ICIs; potential underestimation of PD-L1 by IHC; targeting glycosylation can sensitize tumors to therapy	70-74
Phosphorylation	GSK3β; β-TrCP; AMPK, CK2, NEK2	Growth factor and oncogenic pathways; cytokine signaling; metabolite	Phosphorylation creates a phosphodegron recognized by β-TrCP, triggering ubiquitination and proteasomal degradation; glycosylation at N192/N200/N219 antagonizes this phosphorylation	Limits PD-L1 abundance when GSK3β is active; inactivation of GSK3β or disruption of the phosphodegron stabilizes PD-L1 and promotes immune escape	52, 75-78
Ubiquitination	β-TrCP, SPOP, HRD1; CSN5, USP19, OTUB1.	Oncogenic and inflammatory pathways; DNA damage; ER stress; upstream PTMs	K48-linked ubiquitination targets PD-L1 for proteasomal (and in some contexts lysosomal) degradation; deubiquitination by CSN5/USP19/OTUB1, counteracts degradation and stabilizes PD-L1	Dynamic control of PD-L1 turnover; E3 activation generally reduces PD-L1 and may sensitize tumors to ICIs/chemo; DUB upregulation stabilizes PD-L1 and promotes immune escape and therapy resistance	76, 77, 79-83
Acetylation	p300; HDAC2; SIRT1/NAMPT axis	Oncogenic and stress signaling; chromatin and NAD⁺ metabolism; cytokines; therapy exposure	Acetylation at K263 by p300 restrains nuclear import; deacetylation by HDAC2 enables interaction with HIP1R/vimentin and importin-α/β, promoting nuclear translocation; may compete with ubiquitination at the same Lys	Nuclear PD-L1 supports transcriptional regulation of immune/stress genes and DNA repair; HDAC2 inhibition blocks nuclear entry and enhances anti-PD-1 efficacy; altered acetylation status contributes to chemo-/radioresistance	10, 84
Lactylation	p300; HDAC2	Elevated lactate in microenvironment; glycolytic reprogramming; Warburg metabolism	Lactylation of K189 modulates PD-L1's interaction network and metabolic signaling; delactylation by HDAC2 promotes vimentin-mediated nuclear translocation and alters cholesterol synthesis	Couples metabolic state to PD-L1 localization and transcriptional programs; delactylation-driven nuclear PD-L1 can accelerate tumor growth and may influence response to immunotherapy	85
Palmitoylation	ZDHHC3, ZDHHC9	Oncogenic signaling; lipid metabolism;	S-palmitoylation at C272 anchors PD-L1 in specific membrane microdomains, enhances its stability, and shields it from lysosomal degradation and certain E3 ligases; depalmitoylation or DHHC inhibition promotes PD-L1 turnover	Increases surface PD-L1 and immunosuppressive capacity; pharmacologic or genetic inhibition of ZDHHC3/9 reduces PD-L1 stability, enhances T-cell activity and improves ICI efficacy in preclinical models	86

**Table 2:** Subcellular localization of PD-L1, key regulators, functions, and biological consequences

Subcellular compartment	Binding partners/regulators	Functions of PD-L1	Biological consequences	References
ER/Golgi	STT3A, B3GNT3 and other N-glycosyltransferases; chaperones; ERAD components (e.g., HRD1); Sigma1, FKBP51s	Co-translational insertion and N-glycosylation of PD-L1 (N35, N192, N200, N219); quality control, folding and maturation; ER-associated degradation of misfolded PD-L1	Determines total PD-L1 production and stability; supports high, sustained surface PD-L1 and immune evasion; altered ER stress/ERAD can either enhance or limit PD-L1-mediated resistance	53, 72, 74, 80
Plasma membrane	PD-1 on T cells; CD80 and other co-receptors; β2M/MHC-I; downstream PD-1 effectors (SHP1/2)	Canonical immune checkpoint function: PD-L1-PD-1 engagement at the immune synapse; recruitment of SHP1/2; inhibition of TCR and co-stimulatory signaling; induction of T cell dysfunction and exhaustion	Local immune evasion; dependence of response to anti-PD-1/PD-L1 antibodies on surface PD-L1 abundance and spatial distribution; partial restoration of T cell function with ICIs	26-29
Early/recycling endosomes	CMTM6, CMTM4, Rab GTPases, ESCRT components	Endocytosis and recycling of PD-L1; protection from lysosomal degradation; stabilization of PD-L1 at the plasma membrane through repeated recycling	Maintains high and persistent surface PD-L1 even under fluctuating stimuli; contributes to resistance to ICIs by preserving a large, recycling-competent PD-L1 pool	47, 75
Late endosomes lysosomes	HIP1R; ESCRT machinery (HRS, ALIX); lysosomal proteases	Sorting of internalized PD-L1 toward degradation; lysosomal breakdown of PD-L1; balance between recycling versus destruction	Fine-tunes PD-L1 turnover and surface density; pharmacologic or genetic disruption of this axis can either enhance PD-L1 degradation (sensitizing tumors to ICIs) or stabilize PD-L1 and promote resistance	10, 84, 122
Cytoplasm	Components of PI3K-AKT-mTOR, TGF-β-Smad and JAK-STAT pathways; autophagy regulators	Integration of growth factor, oncogenic and inflammatory signals; support of survival and proliferation pathways; modulation of metabolic programs (e.g., glycolysis, lactate production); crosstalk with autophagy	Promotes tumor cell survival, proliferation and adaptation under stress; links inflammatory and oncogenic signaling to PD-L1-driven immune-independent progression and chemoresistance	100, 101, 103, 105
Nucleus	HDAC2; HIP1R; vimentin; importin-α/β; DNA damage-response and repair complexes; transcriptional cofactors/transcription factors	Nuclear translocation of PD-L1 via HDAC2-HIP1R-vimentin-importin-α/β axis; association with chromatin and transcriptional machinery; regulation of immune, stress-response and DDR genes; participation in DNA damage sensing/repair	Enhances DNA repair capacity and tolerance to genotoxic stress; promotes radio- and chemoresistance; may reshape the tumor transcriptome toward immune evasion and malignant progression	10, 74, 84, 85, 107
Mitochondria	PINK1, ATAD3A (Parkin and other mitophagy factors, context-dependent)	Localization of PD-L1 to the outer mitochondrial membrane; regulation of mitophagy and mitochondrial quality control; adjustment of oxidative phosphorylation versus glycolysis	Supports metabolic adaptation and survival under hypoxia, oxidative stress or therapy; contributes to resistance to chemotherapy, radiotherapy and possibly ICIs through improved mitochondrial fitness	55
Extracellular vesicles	ESCRT machinery (HRS, ALIX); tetraspanins and vesicle biogenesis factors; cargo adaptors	Packaging of PD-L1 into EVs and other extracellular vesicles; secretion of PD-L1-positive vesicles into circulation and tumor microenvironment	Mediates systemic and long-range immunosuppression beyond the primary tumor site; supports pre-metastatic niche formation; provides soluble/EVs PD-L1 as a potential non-invasive biomarker of disease burden and ICI response	56, 57
